# Antibacterial potential of lactic acid bacteria isolated from raw cow milk in Sylhet district, Bangladesh: A molecular approach

**DOI:** 10.1002/vms3.1463

**Published:** 2024-04-24

**Authors:** Mashuka Nahida Asha, Md. Shahidur Rahman Chowdhury, Hemayet Hossain, Md. Anisur Rahman, Ahsan Al Emon, Fatema Yeasmin Tanni, Md. Rafiqul Islam, Md. Mukter Hossain, Md. Mahfujur Rahman

**Affiliations:** ^1^ Department of Medicine Faculty of Veterinary Animal and Biomedical Sciences Sylhet Agricultural University Sylhet Bangladesh; ^2^ Department of Anatomy and Histology Faculty of Veterinary Animal and Biomedical Sciences Sylhet Agricultural University Sylhet Bangladesh

**Keywords:** antibacterial, lactic acid bacteria, *Lactobacillus*, polymerase chain reaction

## Abstract

**Background:**

The most prevalent probiotic bacterium employed in the food industry is *Lactobacillus* because it can produce metabolites with antibacterial capabilities and exhibits hostility towards infections and microorganisms that cause spoilage.

**Aim:**

This study set out to identify naturally occurring *Lactobacillus* and plantaricin *(pln EF)* coding genes in raw cow milk and to assess the antibacterial potency of isolated *Lactobacillus* isolates.

**Methods:**

Following enrichment in De Man, Rogosa and Sharpe (MRS) broth, single colonies were isolated, and pure colonies were obtained by streaking on MRS agar. The *16S rRNA* gene was amplified using polymerase chain reaction (PCR) to confirm the cultural positivity of all isolates. Additionally, the presence of plantaricin was verified by targeting the *pln EF* gene through PCR.

**Outcome:**

Out of the 166 raw milk specimens acquired from cows, 153 (91.17%; CI: 86.98–95.76) were identified as positive for *Lactobacillus* through both culture and biochemical screening. Subsequently, 121 (72.89%; CI: 65.46–79.49) of the isolates were affirmed to harbour *Lactobacillus* through PCR analysis. Within this subset, 6 isolates (4.96%; CI: 1.84–10.48) were found to possess the *plnEF* gene. When exposed to Lactobacillus isolates, *Salmonella Typhimurium* and *Salmonella enterica* displayed an average maximum zone of inhibition with a diameter measuring 24 mm. In contrast, *Escherichia coli* exhibited an average minimum zone of inhibition, featuring a diameter of 11 mm. Additionally, the *Lactobacillus* isolates demonstrated inhibitory zones against *Staphylococcus aureus, Klebsiella pneumoniae* and *Klebsiella oxytoca*, measuring 14, 22 and 19 mm, respectively.

**Clinical significance:**

Lactic acid bacteria, particularly Lactobacilli, are plentiful in cow milk and possess broad‐spectrum antibacterial properties.

## Graphical abstract

Lactic acid bacteria specifically Lactobacillus, abundantly present in cow milk, have demonstrated significant broad‐spectrum antibacterial efficacy against multidrug‐resistant bacteria, particularly through the expression of plantaricin (*pln EF*) coding genes. The occurrence of *lactobacillus spp*. was 72.89%, with the *plnEF* coding gene detected in 4.96%. Lactobacillus isolates carrying the *plnEF* gene exhibited significant inhibition zones against *Salmonella Typhimurium* (24 mm) and *Salmonella enterica* (24 mm), *Escherichia coli* (11 mm), *Staphylococcus aureus* (14 mm), *Klebsiella pneumoniae* (22 mm) *and Klebsiella oxytoca* (19 mm).

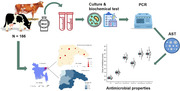

## 1 INTRODUCTION

Disease has always posed a significant challenge in the livestock sector, adversely affecting animal health and welfare. The emergence of antimicrobial resistance among pathogenic bacteria in both humans and animals has become a pressing concern in recent times. Historically, chemotherapeutic drugs and antibiotics supplemented in animal feed as growth promoters have been used to combat and prevent diseases. However, to counteract the development of antibiotic resistance, several countries have implemented restrictions or bans on the use of antibiotics in animal feed. Consequently, the livestock industry is now compelled to seek alternatives to antibiotics for growth promotion and prophylaxis (Manyi‐Loh et al., 2018). One area gaining increasing attention is the development of novel probiotic‐based foods. Probiotic foods are anticipated to comprise around 60%–70% of the functional food industry (Ashaolu, 2021; Kołozyn‐Krajewska & Dolatowski, 2012). Lactic acid bacteria (LAB) is considered the most significant group of probiotic bacteria used in processed dairy products, particularly in milk, their natural habitat (Ahansaz et al., 2023; Delavenne et al., 2012; Wouters et al., 2002).

Lactobacilli, being one of the most important and robust probiotic bacteria, produce antimicrobial peptides and volatile organic acids, rendering them inherently resistant to most antibiotics (Saadatzadeh et al., 2013). Bacteriocin, an antimicrobial peptide produced by LAB, holds the potential to eliminate phylogenetically related strains and prevent spoilage without heat treatment (Simons et al., 2020). In the face of antimicrobial resistance posing a significant threat to animals and human alike, bacteriocins may serve as a natural alternative to antibiotics (Gradisteanu Pircalabioru et al., 2021). Moreover, there has been growing interest in using direct‐fed microbes in animal feed as potential substitutes for antibiotics and growth promoters (McAllister et al., 2011).

In monogastric animals, LAB probiotics are employed to stabilize gut flora, whereas in ruminants, they are used to stabilize the ruminal environment and for biological therapies under Generally Recognized as Safe guidelines (Bhogoju & Nahashon, 2022). *Lactobacillus*, found in milk and other dairy products, has demonstrated its ability to enhance nutrient bioavailability, and its metabolites can serve as preservatives (Ayivi et al., 2020). Over the past few decades, numerous *Lactobacillus* species have been incorporated into a wide range of food products to enhance their nutritional value for consumption by both human and animals (Giraffa et al., 2010). Furthermore, the identification of *Lactobacillus* strains are crucial due to the strain‐specific characteristics of probiotics, the need for quality control of certified strains to prevent health risks and misleading claims, and the requirement to describe new strains (Markiewicz et al., 2010). Molecular techniques, such as DNA‐DNA hybridization, DNA sequence analysis and polymerase chain reaction (PCR) tests, have been developed to accurately identify lactobacilli (Huang et al., 2018). Phylogenetic analysis using *16S rRNA* gene sequences, along with genotypic and phenotypic comparisons with strains stored in databases, is widely employed for species‐level identification of *Lactobacillus* (Patel et al., 2012).

In Bangladesh, researchers have characterized and evaluated LAB from dahi, milk, cheese and yogurt for their potential probiotic properties in districts, such as Dhaka, Chattogram and Jashore (Afrin et al., 2021; Reuben et al., 2020; Shahriar et al., 2019). However, the molecular detection of *Lactobacillus* or probiotics present in raw cow milk, along with their antimicrobial performance, has not been extensively studied in Bangladesh. Therefore, the objective of the present study was to investigate the molecular detection of the *Lactobacillus* genus, natural probiotic presence and *pln EF* (plantaricin) coding genes in raw milk from cows. Additionally, the study aimed to evaluate the antibacterial activity of *Lactobacillus* isolates.

## 2 MATERIALS AND METHODS

### 2.1 Study area and sampling

**FIGURE 1:**
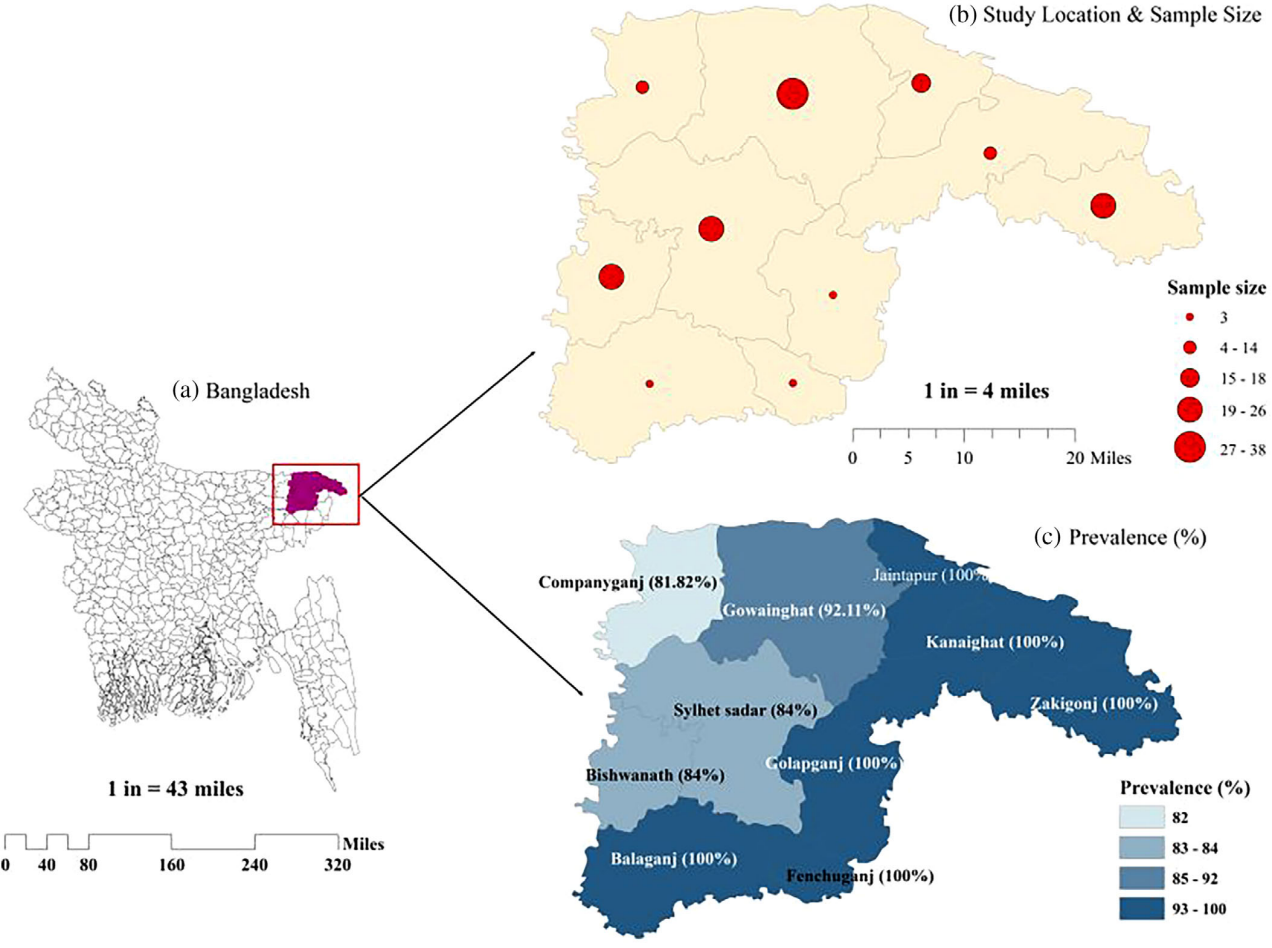
Geo‐spatial mapping of study area showing specific study location, sample size and location‐based prevalence. (a) Geographical area of Bangladesh (b) Selected study area

The study was conducted in various government and private dairy farms in Sylhet, located at 24°36′–25°11′ north latitudes and 91°38′–92°30′ east longitudes, covering an area of 3452.07 km^2^ (Figure 1). A total of 166 milk samples were collected from local and cross‐breed dairy cattle in different dairy farms in Sylhet, selected through simple random sampling. Data collection during sampling was performed using a well‐structured questionnaire. The study was carried out between November 2021 and April 2022.

### 2.2 Bacteria isolation and biochemical characterization

In a test tube, 9 mL of MRS (De Man, Rogosa and Sharpe) broth (Hi‐Media) was combined with 1 mL of milk sample and incubated at 37°C for 24 h to observe the bacterial culture. Primary culture colonies were sub‐cultured on MRS agar (Hi‐Media) and incubated at 37°C for 24 h under strict microaerophilic conditions. Large white colonies from the MRS agar were sub‐cultured into new MRS agar plates. The cultures were streaked on MRS agar twice to obtain pure cultures. Finally, desired bacterial colonies from the pure culture were collected for morphological characterization, biochemical tests and DNA extraction. The remaining colonies were stored in BHI (brain heart infusion) broth and 15% glycerin for future use. The chemical nature of the bacterial isolates was assessed through biochemical tests, including the catalase test, methyl red test, citrate utilization test and carbohydrate fermentation tests, following standard protocols.

### 2.3 Salt (NaCl) tolerance test

*Lactobacillus* isolates were tested for their salt tolerance (Soni et al., 2021) in MRS broth containing different concentrations of NaCl (2%, 4% and 6.5%). Fresh cultures were inoculated into the salt‐containing MRS broth and incubated for 48 h at 37°C. Un‐inoculated MRS broth was used as a control. The presence of turbidity indicated tolerance to salt.

### 2.4 Molecular detection of genus *Lactobacillus* and *PlnEF* gene

The genomic DNA of the entire genome was extracted using the Add Prep Genomic DNA Extraction kit, following the manufacturer's instructions (AddBio Inc. Ltd.). For the amplification of the *16S rRNA* gene of *Lactobacillus* species, universal primers 27F (5‐AGAGTTTGATCCTGGCTCAG‐3′) and 1392R (5‐GGTTACCTTGTTACGACTT‐3′) were used, resulting in a product size of 1350 bp (Saeed et al., 2020; Vasudha et al., 2023). Conventional PCR was performed using DreamTaq Green PCR Master Mix (2X) (Thermo Fisher Scientific), following the manufacturer's instructions. The PCR reaction mixture (20 μL) contained 1 μL (10 pmol/μL) of each forward and reverse primer, 5 μL of the DreamTaq Green PCR Master Mix (2X) and 8 μL of nuclease‐free water. Finally, 5 μL of the DNA template was added to each reaction tube. The amplification conditions included an initial denaturation (94°C for 3 min), followed by denaturation, annealing and extension (35 cycles at 94°C for 45 s, 56°C for 45 s and 72°C for 45 s), and a final extension at 72°C for 7 min.

Similarly, for the *Pln EF* gene amplification, the primers F (5′‐GGCATAGTTAAAATTCCCCCC ‐3′) and R (5′‐CAGGTTGCCGCAAAAAAAG‐3′) were used, resulting in a 428‐bp fragment (Refay et al., 2020). The PCR reaction mixture (25 μL) contained 0.5 μL (10 pmol/μL) of each forward and reverse primer, 12.5 μL of GoTaq Promega Green Master Mix (2X) (Promega Corporation, 2800 Woods Hollow Road Madison) and 8.5 μL of nuclease‐free water. Finally, 3 μL of the DNA template was added to each reaction tube. The amplified PCR products were subjected to electrophoresis in a 1.5% agarose gel (Sambrook et al., 1989). A 100‐bp DNA ladder (KAPA Universal DNA Ladder, cat # KK6302) was used as a molecular weight marker, and the gels were stained with safe gel stain dye, examined, and photographed under a UV transilluminator (Vilber‐Lourmater UV light EEC/France). Positive amplification for the universal *16S rRNA* gene and *Pln EF* gene was confirmed by observing fragment sizes of approximately 1350 and 428 bp, respectively.

### 2.5 Antimicrobial performance of *Lactobacillus* isolates against food‐borne ESBL producing organisms

The antibacterial potential of *Lactobacillus* isolates was evaluated against multidrug‐resistant and extended spectrum beta‐lactamase (ESBL) producing strains of *Klebsiella pneumoniae, Klebsiella oxytoca, Escherichia coli, Staphylococcus aureus, Salmonella Typhimurium* and *Salmonella enterica* using an agar well diffusion assay. After preparing Mueller Hinton Agar plates, PCR‐confirmed *Lactobacillus* isolates were inoculated into MRS broth and incubated at 37°C for 48 h. The grown culture was then preserved as *Lactobacillus* cell culture in an Eppendorf tube. For testing, four or five isolated colonies of the target organisms were suspended in 2 mL of sterile salt solution using a sterile swab. The tube was vortexed to ensure uniform consistency of the suspension. The test organisms were streaked onto the Mueller Hinton Agar plate using the carpet culture technique, ensuring complete coverage of the plate's surface. After allowing the plate to air dry for 5 min, 6 mm diameter wells were created in the agar, and each well was filled with 100 μL of *Lactobacillus* cell culture, whereas the control well contained sterile MRS broth. The plates were then inverted and incubated for 24 h at 37°C. After the incubation period, the zone of inhibition was measured using a metric ruler, considering the diameter of the well in the calculation.

### 2.6 Statistical analysis

Data from the animal sources and laboratory work were entered into a Microsoft Excel spreadsheet. Prevalence was analysed using the following formula:

Prevalence of *Lactobacillus* = (total number of *Lactobacillus*‐positive samples/total number of milk samples) × 100
Prevalence of *Pln EF* gene = (total number of *Pln EF*‐positive samples/total number of *Lactobacillus*‐positive samples) × 100.

Conducted a univariate analysis utilizing the Chi‐square test to assess the associations among various explanatory variables. In cases where the expected count in a cell was less than 5 and occurred in at least 20% of the cells, Fisher's exact test was applied. Confidence intervals were calculated using the Binomial exact test, and a significance level of less than 0.05 was chosen for determining statistical significance. The data analysis was carried out using SPSS version 28 (IBM, 2021).

#### 2.6.1 Geo‐spatial mapping and plot

The study area mapping was generated using ArcMap 10.7 (Vaisi‐Raygani et al., 2021), utilizing a shapefile extracted from (www.diva‐gis.org). This data was employed to create both choropleth and dot maps, effectively visualizing the prevalence of some explanatory variables as well as the corresponding sample sizes. Additionally, to illustrate the antimicrobial properties of *Lactobacillus* isolates, we employed OriginPro (www.originlab.com) (Seifert, 2014) and utilized the Polar Heat Map file exchange format. This allowed to us create informative polar heat maps and mean plots, offering a comprehensive view of the data.

## 3 RESULTS

### 3.1 Results of bacterial isolation and biochemical characterization

#### 3.1.1 Primary isolation of positive samples by culture media

One hundred sixty‐six raw milk samples from apparently healthy cows were analysed for detection of *Lactobacillus* where 153 samples were found positive in primary isolation using culture media. The overall prevalence of *Lactobacillus* was found 91.17% (Table 1). *Lactobacillus* on MRS agar produced white or creamy yellow single colonies with round edges and smooth surfaces with diameters ranging from 0.5 to 3 mm (Figure S1A–C).

**TABLE 1:** Prevalence of *Lactobacillus* and *Pln EF* gene on milk sample of cattle from Sylhet district.

Category	Explanatory variable	Test (+ve)	Total tested	Prevalence% (95% CI)	*p*‐Value
**Diagnostic test**					<0.0001
	Culture and biochemical	153	166	92.17 (86.98–95.76)	
	PCR	121	166	72.89 (65.46–79.49)	
**PCR findings**					<0.0001
	*Lactobacillus*	121	166	72.89 (65.46–79.49)	
	*Pln EF* gene	6	121	4.96 (1.84–10.48)	
**Location**					0.195^b^
	Balaganj	3	3	100.00 (29.24–100.00)^a^	
	Bishwanath	21	25	84.00 (63.92–95.46)	
	Companyganj	9	11	81.82 (48.22–97.72)	
	Fenchuganj	3	3	100.00 (29.24–100.00)^a^	
	Golapganj	3	3	100.00 (29.24–100.00)^a^	
	Gowainghut	35	38	92.11 (78.62–98.34)	
	Kanaighat	14	14	100.00 (76.84–100.00)^a^	
	Jaintapur	18	18	100.00 (81.47–100.00)^a^	
	Sylhet sadar	21	25	84.00 (63.92–95.46)	
	Zakigonj	26	26	100.00 (86.77–100.00)^a^	
**Lactation**					0.099
	First lactation	25	28	89.29 (71.77–97.73)	
	Second lactation	31	37	83.78 (67.99–93.81)	
	Third lactation	65	67	97.01 (89.63–99.64)	
	Fourth lactation	32	34	94.12 (80.32–99.28)	

Abbreviations: CI, confidence interval; PCR, polymerase chain reaction.

^a^ One‐sided 97.5% confidence interval.

^b^ Fisher's exact test where minimum 20% cell have expected count less than 5.

#### 3.1.2 Results of morphological examination

The bacteria were stained blue and purple, and rod‐like bacilli or sphere‐shaped cocci without spores were visible under the microscope (Figure S1A–C). Gram‐positive microorganisms were presumptively identified as *Lactobacillus*.

#### 3.1.3 Results of biochemical tests

##### 3.1.3.1 Results of catalase test

The absence of bubble indicated that the isolated bacteria lack catalase and consequently in capable of mediating the decomposition of hydrogen per oxide into oxygen (Figure S2A). The catalase test is used to assess whether or not bacteria have the enzyme catalase, which is involved in the conversion of hydrogen peroxide into water and oxygen.

##### 3.1.3.2 Results of carbohydrate fermentation test

All of the isolates produced acid by fermenting the three basic sugars (Lactose, Glucose and Sucrose). The change in colour from reddish to yellow in both slant and butt indicated a drop in the pH because of acid production without the formation of gas or hydrogen sulphide (Figure S2B).

##### 3.1.3.3 Results of citrate utilization test

The isolates were unable to utilize citrate as a solitary source of carbon and were not able to generate sodium bi carbonate or ammonia. Thus, there was no alteration in the colour of media (Figure S2C). All of the isolates were tested negative for citrate.

##### 3.1.3.4 Results of methyl‐red test

The appearance of red or pink colour on the surface medium after adding five drops of methyl red suggested acidity and indicated that the isolates were MR test positive (Figure S2D).

### 3.2 Results of salt (NaCl) tolerance test

In MRS broth containing 2%, 4% and 6.5% NaCl, all of the isolates were able to grow (Table S1).

### 3.3 Overall prevalence of genus *Lactobacillus* and *Pln EF* gene in cow raw milk

**FIGURE 2:**
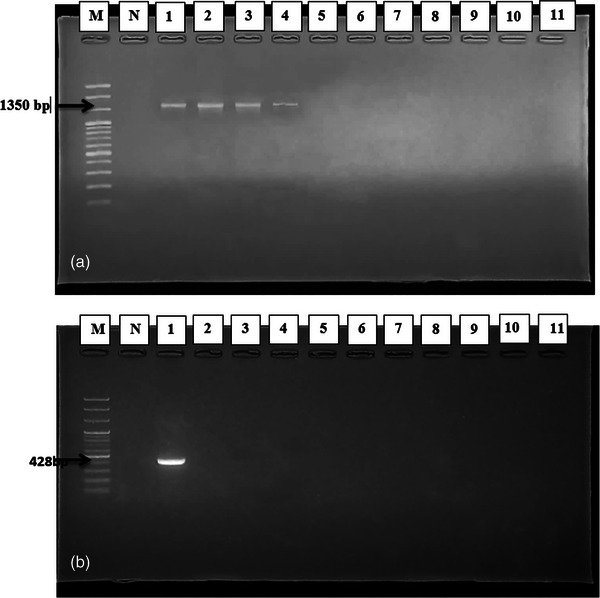
(a and b) Electrophoresis on 1.5% agarose gel showing specific amplified band of *16S rRNA* gene and *Pln EF* gene amplification by polymerase chain reaction (PCR); Lane M: 100 bp Marker DNA; Lane N: Control (−ve); Lane (1–4) reaction specific (+ve) for *16S rRNA* gene (1350 bp) of *Lactobacillus* (a); Lane 1 reaction specific (+ve) for *Pln EF* gene (428 bp) of isolated *Lactobacillus* (b).

The amplified PCR products using appropriate primers were visualized by UV trans‐illuminator. Fragment sizes of approximately 1350 bp for the universal *16S rRNA* gene (Figure 2A) and 428 bp for the *plnEF* gene were confirmed as positive (Figure 2B).

The prevalence was determined on different category based on diagnostic test, PCR findings, location and lactation number, as shown in Table 1. Out of 166 raw milk samples from cow, 153 (91.17%) were found positive for *Lactobacillus* by cultural and biochemical examination (Table 1). Prevalence of *Lactobacillus* in cow raw milk was (72.89%; CI: 65.46–79.49) by PCR assay. From the 121 isolates were confirmed as *Lactobacillus*, 6 tested positive for *plnEF* genes by PCR. The prevalence of bacteriocin protein *plnEF* gene was (4.96%; CI: 1.84–10.48) by PCR assay. Most of the sub‐districts of Sylhet showed 100% positive for *Lactobacillus*, whereas the Companiganj showed the lowest prevalence (81.82%; CI: 48.22–97.72) in Sylhet district. In case of lactation number, the highest prevalence (97.01%; CI: 89.63–99.64) of *Lactobacillus* was observed at third lactation, whereas the lowest prevalence (83.78%; CI: 67.99–93.81) was found on second lactation (Table 1).

### 3.4 Antimicrobial performance

**FIGURE 3:**
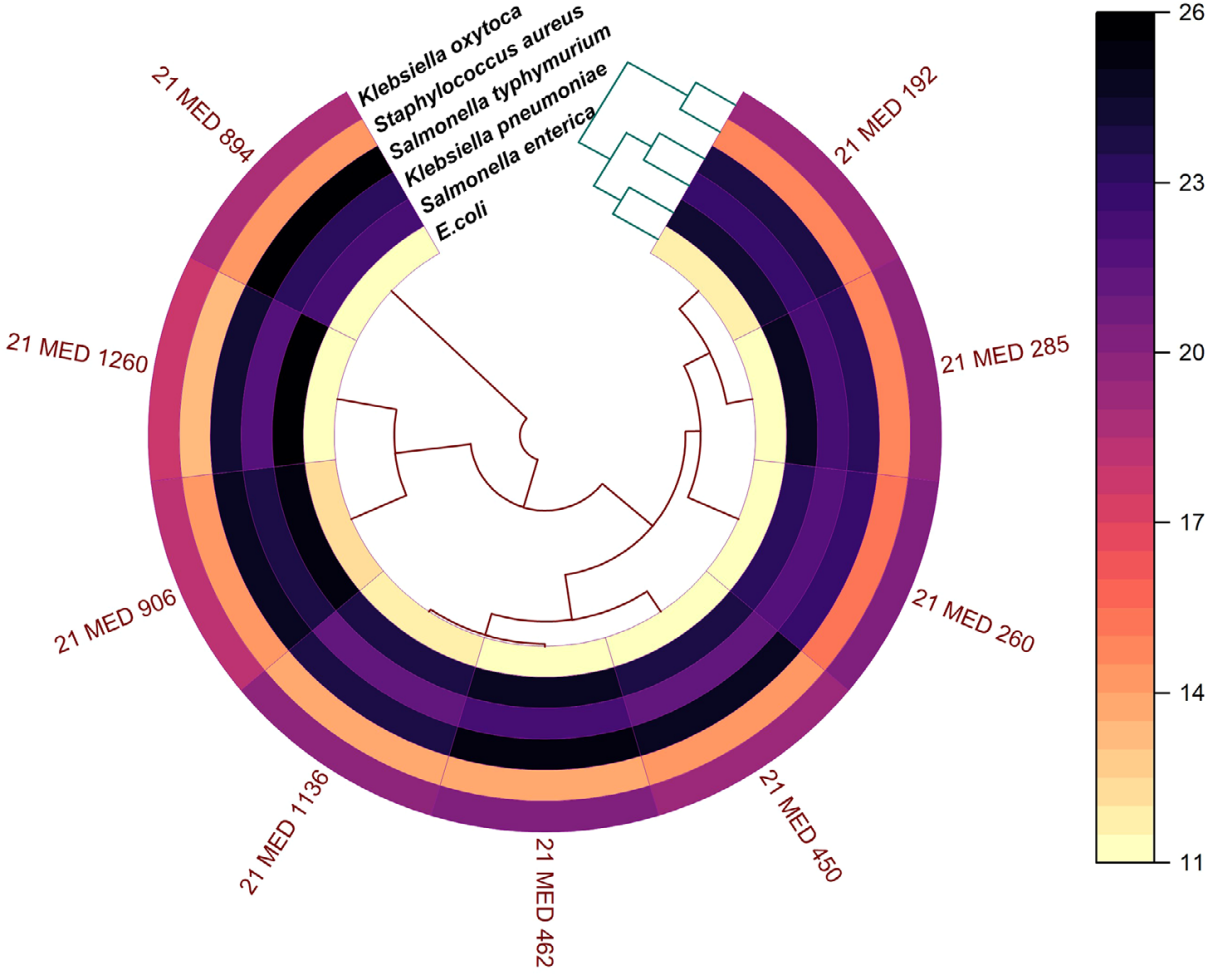
Polar heatmap showing the antimicrobial potency of *Lactobacillus* isolates against selected multi‐drug resistant bacteria.

**FIGURE 4:**
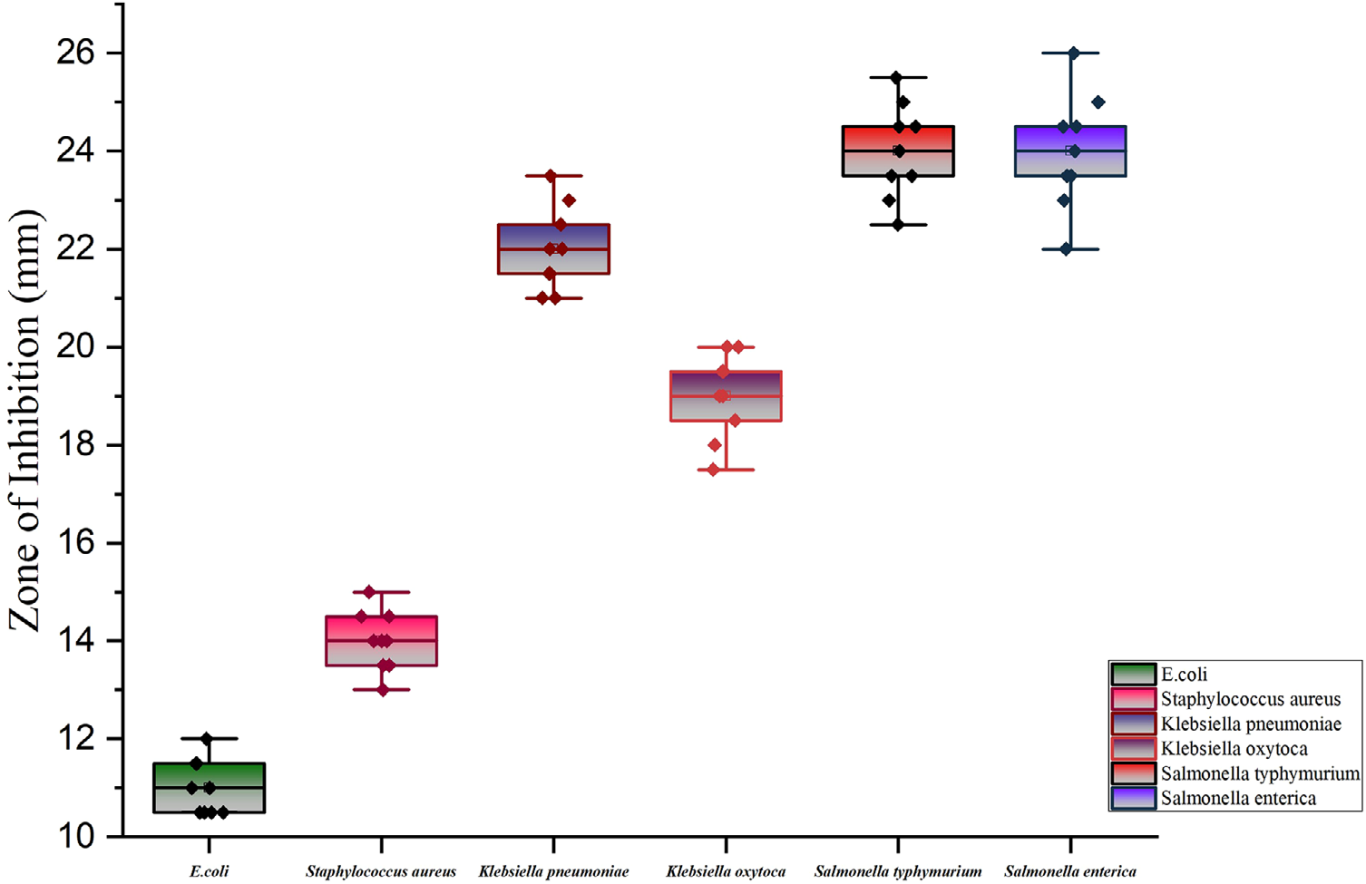
Mean plot showing the antimicrobial activity (average zone of inhibition) against *Escherichia coli*, *Staphylococcus aureus*, *Klebsiella pneumoniae*, *Klebsiella oxytoca*, *Salmonella Typhimurium* and *Salmonella enterica*.

The agar‐well diffusion method was used to assess the antibacterial activity of Lactobacillus isolates against *E. coli*, *S. aureus*, *K. pneumoniae*, *K. oxytoca*, *S. Typhimurium* and *S. enterica*, which are important pathogens for both humans and animals. The zone of inhibition revealed that Lactobacillus isolates possess antibacterial properties against the examined pathogens (Figure 3). The isolated Lactobacillus exhibited its most significant antibacterial activity with an average inhibition zone of 24 mm against *S. Typhimurium* and 22 mm against *K. pneumoniae*, whereas its lowest average inhibition, measuring 11 mm, was observed against *E. coli* (Figure 4).

## 4 DISCUSSION

According to the results of this study, *Lactobacillus* colonies produced a diameter of 0.5–2 mm with a white to yellowish white colour and round edges on MRS agar when incubated at 37°C, which is consistent with the findings of a previous study where milky white round colonies were also observed on MRS agar (Jose et al., 2015). In a separate study (Hoque et al., 2010), *Lactobacillus* from yogurt samples was examined for morphology, as well as various biochemical and physiological properties. Under the microscope, gram‐positive rods or sphere‐shaped bacteria were observed. The isolated bacteria were catalase negative, citrate negative and MR positive. Fermentation of lactose, glucose and sucrose was also observed in *Lactobacillus* isolates, which aligns with the findings of Hoque et al. (2010). Similar morphological and biochemical characteristics of LAB isolated from dahi samples were reported in the study by Harun‐Ur‐Rashid et al. (2007). The use of MRS medium for initial identification of the *Lactobacillus* genus was chosen due to the inability of other species to grow in this medium, which is in agreement with another report suggesting the prevalence of the *Lactobacillus* genus in MRS medium (López‐Díaz et al., 2000; Vasudha & Gayathri, 2023).

In the present investigation, the prevalence of *Lactobacillus* isolates in raw cow milk was assessed using both cultural and biochemical methods, revealing a rate of 91.07%. However, when employing PCR, the prevalence was slightly lower at 72.02%. These results align with those reported by Abdullah and Osman, who conducted a study on the prevalence of the *Lactobacillus* genus in Sudanese fermented milk (rob), raw milk and white cheese. In their research, the prevalence, determined through cultural, physiological and biochemical tests, was reported to be 69.23% (Abdullah & Osman, 2010). In a separate investigation (Saeed et al., 2020), the prevalence of *Lactobacillus* in raw goat milk was determined to be 15% through PCR analysis. This suggests a higher incidence of probiotic *Lactobacillus* strains in cow milk when compared to goat milk. Furthermore, when examining the prevalence of the *Lactobacillus* genus in raw milk, cheese and yogurt using cultural, physiological and biochemical tests, the reported rate was 24.38% (Taye et al., 2021). It is noteworthy that this prevalence is comparatively lower than the findings observed in the current study. Moreover, the prevalence of the bacteriocin protein *plnEF* gene in this research was determined to be 4.96%, which stands in stark contrast to a prior study in Egypt (Refay et al., 2020). In their investigation, the prevalence of the *plnEF* gene was reported to be substantially higher at 32.35% (22/68).

Antibacterial activity is crucial for Lactobacilli colonization in the intestinal mucosa as it acts as a barrier and provides protection against pathogens (Dempsey & Corr, 2022). *Lactobacillus* produces antimicrobial components, such as organic acids, hydrogen peroxide, diacetyl and bacteriocins – low molecular weight antimicrobial compounds – that exhibit inhibitory effects against pathogens (Santos et al., 2003). Due to concerns such as the emergence of resistant bacteria and the presence of residual antibiotics in livestock products, the usage of antibiotics in animal feed needs to be controlled, and organic approaches for livestock rearing have been recommended (Vanderhaeghen & Dewulf, 2017). In this study, the *Lactobacillus* isolates demonstrated antibacterial activity and inhibited the growth of the tested pathogens. These findings are consistent with other studies that suggest *Lactobacillus* species as a common probiotic bacteria used as an alternative measure to prevent Salmonella‐related diseases (Kowalska et al., 2020). Similarly, Djadouni and Kihal (2012) reported that LAB produce antibacterial compounds that inhibit the growth of indicator organisms, resulting in inhibition zones of 10–14 mm in diameter against *E. coli, S. aureus* and *S. Typhimurium* using the agar spot test. The antagonistic activity of LAB isolates against *Salmonella typhi, K. pneumoniae* and *S. aureus* observed in this study was similar to the findings of (Prabhurajeshwar & Chandrakanth, 2017). The antibacterial capability of the isolates in this study was comparable, and the fact that *Lactobacillus* isolates exhibited antibacterial activity against both gram‐positive and gram‐negative organisms demonstrates their broad‐spectrum activity.

## 5 CONCLUSION

*Lactobacillus* is a probiotic with various applications for both human and animal health. In this study, the prevalence of *Lactobacillus* in raw cow milk was found to be 72.89%, whereas the prevalence of the *pln EF* gene was determined to be 4.96% using PCR assay. The LAB isolates exhibited significant inhibition zones against *S. Typhimurium* and *S. enterica*, with an average maximum diameter of 24 mm, and against *E. coli*, with an average minimum diameter of 11 mm. *Lactobacillus* isolates also demonstrated zone of inhibition against *S. aureus, K. pneumoniae* and *K. oxytoca*, with diameters of 14, 22 and 19 mm, respectively. These findings indicate the antibacterial capability of the LAB isolates.

Due to the emergence of antibiotic resistance, finding alternatives to antibiotics for growth promotion and disease prevention is crucial in the livestock industry. When considering *Lactobacillus* isolates, additional requirements must be met beyond antibacterial traits, as *Lactobacillus* has the potential to transfer antibiotic resistance genes to other species. Further focused research, including in vitro and in vivo investigations, animal model studies and human trials, is necessary to ensure the safety and efficacy of clinical applications involving *Lactobacillus*. Additionally, the discovery and characterization of new *Lactobacillus* species strains that offer potential benefits for human and animal health require further study. *Lactobacillus* and bacteriocin molecules may play even more intriguing roles in the future, such as antiquorum sensing and targeted drug delivery.

## AUTHOR CONTRIBUTIONS

*Data curation; formal analysis; investigation; methodology; resources; software; validation; visualization; writing – original draft*: Mashuka Nahida Asha, Md. Anisur Rahman, Ahsan Al Emon, Fatema Yeasmin Tanni and Md. Rafiqul Islam. *Data curation; formal analysis; investigation; methodology; resources; software; validation; visualization; writing – original draft; writing – review and editing*: Md. Shahidur Rahman Chowdhury, Hemayet Hossain and Md. Mukter Hossain. *Conceptualization; data curation; formal analysis; investigation; methodology; project administration; resources; supervision; software; validation; visualization; writing – original draft; writing – review and editing*: Md. Mahfujur Rahman.

## CONFLICT OF INTEREST STATEMENT

The authors declare no conflicts of interest.

## FUNDING INFORMATION

Funding none.

### PEER REVIEW

The peer review history for this article is available at https://publons.com/publon/10.1002/vms3.1463.

## ETHICS STATEMENT

In order to ensure the wellbeing of the animal and to limit animal use on meritorious research the ‘Animal Experimentation and Ethics Committee (AEEC)’, Sylhet Agricultural University, Bangladesh assessed and approved this experiment. This experiment holds an approved Animal Use Protocol [#AUP2022038] for conducting the experiment. The memo no: [SAU/Ethical committee/AUP/22/38].

## Supporting information

Supporting Information

## Data Availability

Data available on request from the corresponding author.
