# Supplementary material for: Antibacterial potential of lactic acid bacteria isolated from raw cow milk in Sylhet district, Bangladesh: A molecular approach
Source: Vet Med Sci. 2024 Apr 24;10(3):e1463. doi: 10.1002/vms3.1463 (PMC11043669; doi:10.1002/vms3.1463)
Supplement: Supplementary file 1 — Supporting Information [file VMS3-10-e1463-s001.docx]

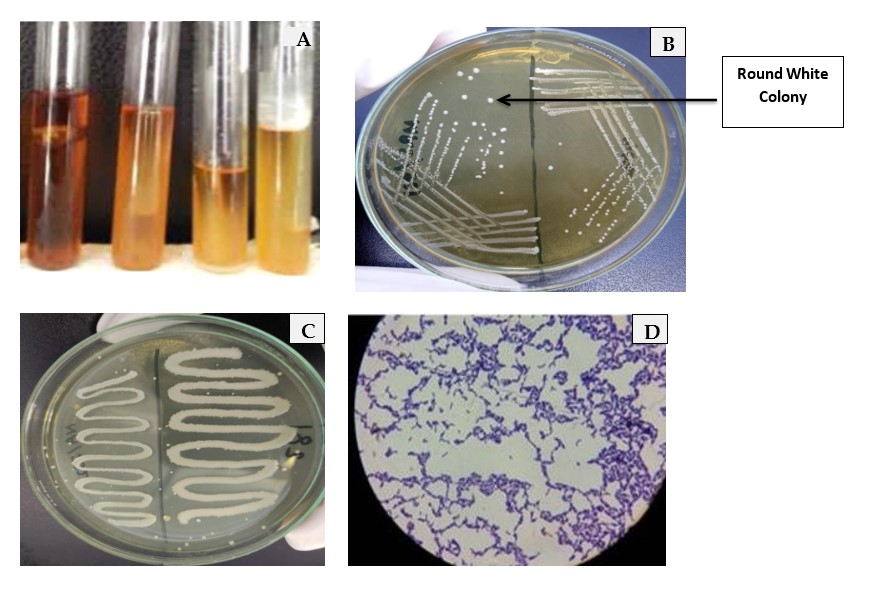


**Supplementary Figure 1(A-D)** Morphological characteristics of *Lactobacillus*: A) Pre-enrichment in MRS broth produced turbidity; B) On MRS agar plate *Lactobacillus* produced white round edge colony; C) Pure culture on nutrient agar; D) Gram positive, purple-blue color, *Lactobacillus* in Gram’s staining under microscope

**
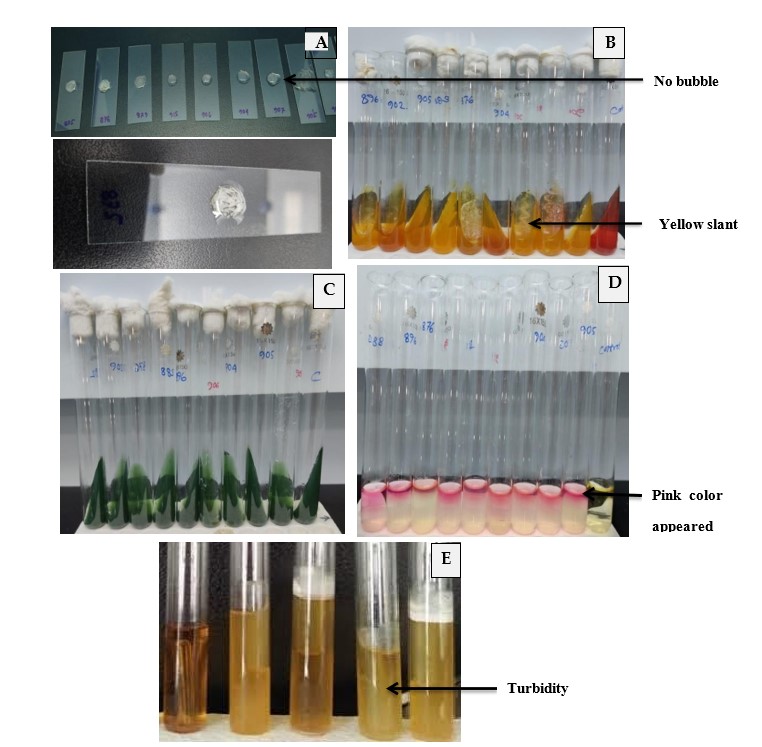
**

**Supplementary Figure 2(A-E)** Biochemical and physiological characteristics of *Lactobacillus:* A) In Catalase test *Lactobacillus* produced no bubble; B) In CHO fermentation test by TSI agar *Lactobacillus* produced yellow color slant and butt; C) In citrate utilization test by Simmons citrate agar *Lactobacillus* did not utilize citrate; D) In MR test *Lactobacillus* produced stable red color; E) In salt tolerance test bacterial growth indicates turbidity.

**Supplementary Table 1:** Results of different biochemical tests of *Lactobacillus* isolates from cow raw milk

| SL. No | Name of tests | Result | Interpretation |
| --- | --- | --- | --- |
| 1. | Catalase test | No bubbles | Catalase (-) ve |
| 2. | Carbohydrate fermentation by TSI agar | Yellow color slant and butt | TSI (+) ve |
| 3. | Citrate utilization test by Simmons citrate agar | No change in the color | Citrate (-) ve |
| 4. | Methyl red test | Red or pink color on the surface medium | MR (+) ve |
| 5. | Salt (Nacl) Tolerance Test | Turbidity | Bacterial growth (+) ve |

TSI = Triple Sugar Iron agar, MR = Methyl red
